# Efficacy of antifungal agents against fungal spores: An in vitro study using microplate laser nephelometry and an artificially infected 3D skin model

**DOI:** 10.1002/mbo3.1257

**Published:** 2022-01-13

**Authors:** Sarah Fink, Anke Burmester, Uta‐Christina Hipler, Claudia Neumeister, Marcus R. Götz, Cornelia Wiegand

**Affiliations:** ^1^ Department of Dermatology University Hospital Jena Jena Germany; ^2^ Dr. Pfleger Arzneimittel GmbH Bamberg Germany

**Keywords:** antifungal, ciclopirox olamine, dermatophyte, sertaconazole nitrate, sporicidal, terbinafine

## Abstract

Dermal fungal infections seem to have increased over recent years. There is further a shift from anthropophilic dermatophytes to a growing prevalence of zoophilic species and the emergence of resistant strains. New antifungals are needed to combat these fungi and their resting spores. This study aimed to investigate the sporicidal effects of sertaconazole nitrate using microplate laser nephelometry against the microconidia of *Trichophyton*, chlamydospores of *Epidermophyton*, blastospores of *Candida*, and conidia of the mold *Scopulariopsis brevicaulis*. The results obtained were compared with those from ciclopirox olamine and terbinafine. The sporicidal activity was further determined using infected three‐dimensional full skin models to determine the antifungal effects in the presence of human cells. Sertaconazole nitrate inhibited the growth of dermatophytes, molds, and yeasts. Ciclopirox olamine also had good antifungal activity, although higher concentrations were needed compared to sertaconazole nitrate. Terbinafine was highly effective against most dermatophytes, but higher concentrations were required to kill the resistant strain *Trichophyton indotineae*. Sertaconazole nitrate, ciclopirox olamine, and terbinafine had no negative effects on full skin models. Sertaconazole nitrate reduced the growth of fungal and yeast spores over 72 h. Ciclopirox olamine and terbinafine also inhibited the growth of dermatophytes and molds but had significantly lower effects on the yeast. Sertaconazole nitrate might have advantages over the commonly used antifungals ciclopirox olamine and terbinafine in combating resting spores, which persist in the tissues, and thus in the therapy of recurring dermatomycoses.

## Graphical abstract

Dermal fungal infections have increased over recent years. This study aimed to investigate the sporicidal effects of antimycotics using microplate laser nephelometry and infected three‐dimensional full skin models. Sertaconazole nitrate inhibited the growth of dermatophytes, molds, and yeasts. Ciclopirox olamine also had antifungal activity, although, higher concentrations were needed compared to sertaconazole nitrate. Terbinafine was highly effective against most dermatophytes, but higher concentrations were required to kill the resistant strain of *Trichophyton indotineae*.

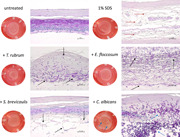

## 1 INTRODUCTION

Dermatomycoses are mostly superficial fungal infections of the skin, hair, and nails caused by dermatophytes, yeasts, and more rarely, molds. Dermatomycoses are one of the most common dermatological conditions, with more than 25% of people affected worldwide, particularly in tropical and subtropical regions (Havlickova et al., 2008). Although dermatomycoses are not life‐threatening, they are considered a public health problem as they affect the quality of life of individuals (Silva et al., 2014) and account for at least half a billion dollars in health costs annually (White et al., 2014). A wide variety of topical and systemic antimycotic agents are available for the treatment of dermatomycoses (Nenoff et al., 2015), including allylamines, such as terbinafine; triazoles, such as fluconazole, itraconazole, voriconazole, and efinaconazole; imidazoles, such as ketoconazole, sertaconazole, and luliconazole; ciclopirox olamine; tavaborole; amorolfine hydrochloride and griseofulvin. The choice of agent depends on the site and the extent of the infection, as well as the causative organism (Gupta et al., 2005; Meis & Verweij, 2001). Topical antimycotics are frequently characterized by a broad therapeutic spectrum that includes dermatophytes, yeasts, and molds, as well as some Gram‐positive bacteria (Nenoff et al., 2015). Superficial mycoses appear to have increased during recent years, although epidemiological data for Germany are scarce as there is no obligation to report these infections and no population‐based epidemiological studies have been carried out. However, from 2007 to 2018, there was a noticeable increase in the number of samples processed in the mycological laboratory of the Department of Dermatology at Jena University Hospital (Wiegand et al., 2019). The turnover and the number of packages of terbinafine and ciclopirox olamine containing prescription and over‐the‐counter medications sold declined significantly in the German market from 2018 to 2020. However, the number of packages containing sertaconazole increased between 2018 and 2019 and declined more moderately than the other two between 2018 and 2020 (Insight Health Database, 2021). Not only the number of fungal infections is steadily rising but there is also a shift from anthropophilic dermatophytes, such as *Trichophyton rubrum* and *Trichophyton interdigitale*, to an increasing prevalence of zoophilic species, like *Trichophyton benhamiae* and *Trichophyton mentagrophytes* (Nenoff et al., 2014). Recently, *T. interdigitale* has been reclassified as an anthropophilic species, and the related zoophilic *T. interdigitale* strain (previously called *T. interdigitale var. mentagrophytes*) has been renamed *T. mentagrophytes* (de Hoog et al., 2017). Part of the *T. mentagrophytes/interdigitale* complex is the new multiresistant species *T. indotineae* (Tang et al., 2021). Resistant *T. indotineae* strains have recently emerged, producing new challenges (Ebert et al., 2020). Consequently, failures of antifungal treatment have been observed, some of which could be related to resistance to the antifungals, such as terbinafine (Burmester et al., 2019; Gupta & Kohli, 2003; Gupta et al., 2021; Mukherjee et al., 2003; Nenoff et al., 2020; Rudramurthy et al., 2018; Salehi et al., 2018; Süß et al., 2019). Point mutations within *Erg1*, which encodes the squalene epoxidase, are responsible for the resistance to allylamines (Burmester et al., 2019, 2020; Ebert et al., 2020; Rudramurthy et al., 2018; Salehi et al., 2018). Interestingly, Ala448Thr *Erg1 T. indotineae* point mutants show also an often increase in azole resistance whereas the molecular mechanism of this linkage remains unresolved (Burmester et al., 2021, 2020; Ebert et al., 2020). Azoles interacting with sterol 14‐α demethylases and point mutations of the corresponding genes is one mechanism to obtain azole resistance (for review Rosam et al., 2021; Song et al., 2018). Recently, point mutations of sterol 14‐α demethylase genes were also identified in azole‐resistant *T. indotineae* strains (Burmester et al., 2021). Another mechanism of azole resistance is the activation of efflux pumps, such as ATP‐binding cassette transporters or major facilitator superfamily transporters (for review Pérez‐Cantero et al., 2020). Activation of efflux pumps was identified in *T. rubrum* as the molecular mechanism of azole resistance (Monod et al., 2019). The persistence of fungal spores could further underlie the failure of antifungal treatments, as well as recurrent infections and chronic diseases. Spores are dormant entities with a minimal metabolism and heavily reinforced cell walls, which make them less susceptible to many therapies (Seidl et al., 2015).

New antifungal agents are needed to combat the range of microorganisms. The antimicrobial activity and effectiveness of these antifungal agents must be characterized, usually using in vitro tests. Such tests enable a direct comparison of the effects of the active substances on microorganisms. They are also simple, fast, reproducible, and inexpensive, as well as able to handle a large number of samples (Wiegand et al., 2015a). Microplate laser nephelometry (MLN) is a method for measuring light that is scattered up to 90° by particles suspended in a solution. It has been successfully employed to monitor the growth of bacteria (Wiegand et al., 2015a, 2015b; Wiegand et al., 2012), yeasts (Finger et al., 2012; Seyfarth et al., 2008), and even dermatophytes (Burmester et al., 2020) by monitoring the turbidity of the medium and to investigate the effect of different substances on the growth of these microorganisms. Compared to other methods used (Suller & Russell, 1999; Walsh et al., 2003), MLN enables high‐throughput screening, long‐term incubation, and in situ monitoring of changes in dose–response curves, as well as the determination of the half‐maximal inhibitory concentration (IC_50_) and minimum inhibitory concentration (MIC).

Active substances are further required to exhibit targeted, antifungal activity in the presence of human cells. In vivo conditions are increasingly being mimicked using three‐dimensional (3D) cell cultures. Such full skin models consist of a collagen matrix populated with primary human fibroblasts as dermis and a fully differentiated epidermis made of primary human keratinocytes (Reddersen et al., 2019; Wiegand et al., 2016). For the investigations in this study, the model was artificially infected with spores to enable the analysis of the sporicidal influence of the substances to be examined under relevant in vivo‐like conditions. Compared to a simple 2D model, the 3D full skin model has the advantage that the cells are in their natural 3D environment and show corresponding behavior. The antifungal and sporicidal effects of various compounds can therefore be examined in situ in the presence of human cells. In addition, the influence on cell viability and the release of lactate dehydrogenase (LDH) over the incubation period was examined. Damage to the cell membrane can be measured by the release of the cytosolic enzyme LDH and can be regarded as a sign of cell necrosis, late apoptosis, and other forms of cellular damage (Wiegand & Hipler, 2009; Wiegand et al., 2010). Because the biocompatibility of the materials is further determined by other cellular and molecular factors, an analysis of cellular interleukin release can help in elucidating proinflammatory effects, which could not be demonstrated by cytotoxicity tests alone (Wiegand & Hipler, 2009; Wiegand et al., 2010). Such mediators are, for example, interleukins‐1α, ‐6, and ‐8, which coordinate cell proliferation, cell migration, and possible inflammatory reactions.

This study aimed to investigate the sporicidal effects of a sertaconazole compound. The effectiveness of sertaconazole nitrate was examined using MLN based on NCCLS M27‐A2, NCCL2 M38‐A, and DIN EN 27027 against the microconidia of *Trichophyton* species, chlamydospores of *Epidermophyton*, blastospores of *Candida* yeasts, and conidia of the mold *Scopulariopsis brevicaulis*. Investigations were limited to one strain each to enable the analysis of a higher number of different species. The results obtained were compared with the antifungal activities of ciclopirox olamine and terbinafine. A 3D full skin model artificially infected with fungal spores was used to demonstrate the sporicidal activity of the substances in situ in the presence of human cells.

## 2 MATERIALS AND METHODS

### 2.1 Antifungal stock solutions and preparation of test solutions

Sertaconazole nitrate (Grupo Ferrer Internacional, S.A.), terbinafine, or ciclopirox olamine (Sigma‐Aldrich; PHB standard quality) were dissolved in dimethyl sulfoxide (Sigma‐Aldrich) at a final concentration of 10 mg/ml to prepare stock solutions. For the determination of antifungal activity, the corresponding test concentrations were prepared by diluting the stock solutions in the Sabouraud glucose medium (Merck). To identify effective concentrations, sertaconazole nitrate was used in the range of 0.001–50 µg/ml, terbinafine concentrations were varied between 0.00005 and 50 µg/ml, and ciclopirox olamine was tested in concentrations ranging from 20 to 100 µg/ml. To investigate the influence of the samples in the infected full skin models, the corresponding test concentrations (Table 1) were prepared by diluting the stock solution in ultrapure water (WFI; Fresenius Kabi).

**Table 1:** Concentrations of sertaconazole nitrate, ciclopirox olamine, and terbinafine for the treatment of infected full skin models

	Sertaconazole nitrate (µg/ml)	Ciclopirox olamine (µg/ml)	Terbinafine (µg/ml)
*Trichophyton rubrum*	25	400	0.050
*Epidermophyton floccosum*	10	400	0.200
*Scopulariopsis brevicaulis*	100	400	100
*Candida albicans*	800	400	1600

### 2.2 Growth of fungal strains

*Candida albicans* DSM 1386, *Candida parapsilosis* DSM 5784, *T. rubrum* DSM 16111, *T. interdigitale* IHEM 22939, *Trichophyton soudanense* DSM 103786, and *Trichophyton indotineae* DSM 107608 were obtained from the DSMZ (German Collection of Microorganisms and Cell Culture) or the Belgian Co‐ordinated Collections of Microorganisms. The isolates *Epidermophyton floccosum* RV51/17 and *S. brevicaulis* RV491II/16 stem from proficiency tests.

Yeasts and molds were cultivated on Sabouraud dextrose agar plates (Merck) for 48 h at 37°C under aerobic conditions. Dermatophytes were grown on Dermasel agar plates (Oxoid) at room temperature for 3 weeks. Preparation of the test suspensions was performed as previously reported (Burmester et al., 2020). In brief, the spores of dermatophytes and *Scopulariopsis* spp. were gently scraped from the agar plate surface and dispersed in 5 ml of sterile isotonic NaCl solution (9 g/L; Fresenius Kabi), which was then filtered through a cell strainer with a mesh size of 40 µm (Greiner Bio‐One). Spore solutions (molds) were counted manually using disposable counting chambers (type Neubauer improved; Carl Roth). *Candida* cell cultures were obtained by inoculation of 20 ml of 2% Sabouraud glucose medium (Merck) with 1–2 colonies and by incubating the culture overnight at 30°C with shaking. Dilutions were made in a 2% Sabouraud glucose medium (Merck) to a final concentration of 2 × 10^3^ spores/ml. The viability of the spores/cells was determined by plating on Sabouraud dextrose agar (Merck). Colonies were counted after 48 h at 37°C (yeast and mold) or up to 7 days of cultivation at room temperature (dermatophytes), and viability was determined as the percentage of the initial concentration.

### 2.3 Determination of the antifungal effect using MLN

The MLN assay was performed as previously described (Burmester et al., 2020) over 48 h (yeast) or 120 h (molds and dermatophytes) at 30°C using NEPHELOstar Galaxy (BMG Labtech). In each case, 100 μl of a dilution was added, together with 100 μl of the microorganism spore suspension, into the well of a sterile, clear 96‐well microplate (Greiner Bio‐One). Blanks for each substance concentration tested were included in each assay. Microtiter plates were closed with a transparent film (Greiner Bio‐One). To enable gas exchange, perforations were made on the right edge of the wells. The microtiter plates were then inserted into the microtiter plate laser nephelometer (NEPHELOstar Galaxy; BMG Labtech). Wells showing complete growth inhibition after the incubation time were plated on the Sabouraud glucose medium to evaluate the fungicidal or fungistatic effects. Colony growth was determined over the following 3 days or up to 7 days depending on the growth behavior of the different species. The absence of colonies was taken as a sign of fungitoxic effects, and it represented the cutoff concentration for the MIC. The IC_50_ of the antifungals under the experimental conditions used was calculated from the growth curves over the incubation time. The area under the curve was determined from the results and calculated as a percentage of the untreated control. This metric was used to produce a dose–response curve for each antifungal tested, from which the IC_50_ was calculated using a logistic fit function [*y* = A2 + (A1 − A2)/(1 + [*x*/x0]*p*); A1 = upper limit (100%), A2 = lower limit (0%), *x*0 = IC_50_, *p* = slope of the curve; Origin Pro 2018G; OriginLab Corporation].

### 2.4 Preparation of the 3D full skin models and infection with the microorganisms

Normal human dermal fibroblasts (Promocell) and normal human epidermal keratinocytes (Promocell) were used to produce the full skin models. Fibroblasts were maintained in Dulbecco's modified Eagle's medium (DMEM; BioConcept) with 2% fetal calf serum (FCS; PAN‐Biotech), 5 ng/ml human fibroblast growth factor (CellSytems), and 5 µg/ml insulin (PeloBiotech), and keratinocytes were cultivated in keratinocyte basal medium (KBM; Promocell). Cell cultivation took place over 7 days in 75 cm² cell culture bottles (Greiner Bio‐One) at 37°C in a 5% CO_2_ atmosphere. The cells were then detached with trypsin‐EDTA (Gibco, Thermo Fisher Scientific) and resuspended in the appropriate medium. To produce the dermis equivalent, fibroblasts were placed in 12‐well inserts (Greiner Bio‐One) with DMEM + 10% FCS + 1% gentamycin (Gibco, Thermo Fisher Scientific) + 150 µg/ml ascorbic acid (Sigma‐Aldrich) and cultivated for 3 weeks at 37°C under 5% CO_2_. The medium was changed every 2–3 days. Before the keratinocytes were seeded to produce the epidermis, the medium surrounding the insert was completely removed, and the top of the dermis was coated with fibronectin (50 μg/ml; Promocell). After 30 min incubation of the dermis equivalents with fibronectin, the keratinocytes were seeded at a density of 2 × 10^5^ cells/insert in KBM + 5% FCS + 1% gentamycin + ascorbic acid (150 µg/ml). The full skin models were then incubated for 45 min in an incubator before being flooded with KBM + 5% FCS + 1% gentamycin + ascorbic acid (150 µg/ml). The skin models were cultivated for 7 days, submerged in the medium with decreasing FCS concentration (5%). To do this, the medium was changed every 2–3 days. After submerged cultivation, the full skin models were cultivated at the air‐medium border (airlift cultivation) for a further 12 days. For this purpose, the full skin models were transferred to ThinCert 12‐well plates (Greiner Bio‐One) and supplemented with Green's medium (1:1 DMEM + DMEM HAMS‐F12 (Gibco, Thermo Fisher Scientific), as previously reported (Reddersen et al., 2019; Wiegand et al., 2016). The medium was changed every 2–3 days. For artificial infection, collagen pellicles were prepared from a 6 mg/ml collagen suspension (Fraunhofer Institute) and loaded with 25 µl of a suspension of spores of the respective microorganisms (10^4^/ml, *C. albicans*: 2 × 10^2^/ml). The suspension was allowed to dry for 1 h. Then skin models were placed on the spore‐loaded collagen pellicles.

### 2.5 Treatment of the infected 3D full skin models

The concentrations of sertaconazole nitrate, ciclopirox olamine, and terbinafine used in the skin model were selected from a preliminary determination of MIC values. To take into account a reduced penetration of the substances through the corneal layer and possible adsorption processes on cells, lipids, and proteins, the antimycotic substances were used in concentrations that had 10 times the determined preliminary MIC values (Table 1). Immediately after placing the skin models on the spore‐loaded collagen pellicles, the Green's medium was replaced by a fresh medium, and 25 μl of the substance solution was applied directly onto the spore‐bearing whole skin models. The negative control consisted of 25 µl of ultrapure water (WFI), and 25 µl of 1% sodium dodecyl sulfate (SDS; Roth) served as a positive control. The test samples were incubated for 48 and 72 h, respectively, at 37°C under 5% CO_2_.

### 2.6 Determination of fungi, cell viability, and cytotoxic effects

Infection of the full skin models was verified using hematoxylin and eosin (HE) staining as well as periodic acid–Schiff (PAS) staining. Therefore, 3D skin models were placed in embedding cassettes in 4% formalin solution (Dr. K. Hollborn & Sons), after which they were drained and paraffinized overnight in a tissue processor Shandon Excelsior ES (Thermo Fisher Scientific). The 3D skin models were then poured into blocks with paraffin (Merck Millipore) and cured. Sections with a thickness of 4 µm were made and mounted on microscope slides. For staining, the paraffin contained in the tissue sections was removed using a descending alcohol series. Dewaxing and staining were carried out automatically using Leica Autostainer XL (Leica Mikrosysteme Vertrieb GmbH) according to the manufacturer's protocol. The stained sections were fixed on the slide with a drop of embedding medium and a coverslip. The microscopic assessment was carried out using an Axio Scope A.1 microscope (Carl Zeiss), and images were taken with an AxioCam MRc digital camera (Carl Zeiss). The images were processed and evaluated using the ImageJ program (National Institutes of Health, 2004). Cell viability was determined based on the luminometric ATP measurement using the ATPLiteTM‐M assay (PerkinElmer) as previously reported (Reddersen et al., 2019; Wiegand et al., 2016). In brief, 500 µl of the cell lysis solution was added to each full skin model. The mixture was then shaken on an orbital shaker at 700 rpm for 15 min, 150 µl was transferred to a white 96‐well microtiter plate, and 50 µl of substrate solution (luciferin/luciferase) was added to each well according to the manufacturer's protocol. Luminescence intensity was measured using a LUMIstar Galaxy microplate luminometer (BMG Labtech). LDH release was measured using cytotoxicity detection kits (Roche Diagnostics) as previously reported (Reddersen et al., 2019; Wiegand et al., 2016). In short, 100 µl of the culture supernatants of the full skin models was transferred in duplicate to a transparent 96‐well microtiter plate (Greiner Bio‐One), and 100 µl of the tetrazolium dye solution was added per well and incubated for 30 min at room temperature in the dark. The absorption at 490 nm was measured using a POLARstar Galaxy photometer (BMG Labtech). The measurement of the interleukin release by the full skin models during treatment with the samples was carried out using the corresponding enzyme‐linked immunosorbent assays (ELISAs) for interleukin 1α (IL‐1α; Bio‐Techne, Bio‐Techne/Headquarters), IL‐6 (Mabtech AB), and IL‐8 (Bio‐Techne). The ELISAs were carried out according to the manufacturer's instructions. The culture supernatants of the whole skin models were used undiluted. The absorption measurements were carried out at 450 nm with a reference wavelength of 620 nm in a POLARstar Galaxy photometer (BMG Labtech). Quantitative evaluation was based on a standard curve.

### 2.7 Statistical analysis

Experiments were carried out in two independent tests. Measurements were performed four times (MLN) or twice (full skin model). The mean values ± standard deviation is given. The statistical evaluation of the measured values was performed using Student's *t*‐tests in Microsoft® Excel SP2. Values of *p* < 0.05 were taken to indicate statistical significance.

## 3 RESULTS

### 3.1 Determination of antifungal activity by MLN

MLN has shown that sertaconazole nitrate efficiently reduces microbial growth. The highest activity determined in vitro was against *T. soudanense*, with an average IC_50_ value of 0.0014 µg/ml, *T. indotineae* with 0.0018 µg/ml, and *T. rubrum* with 0.0028 µg/ml (Table 2, also see Appendix A). Significantly higher amounts of sertaconazole nitrate were required to inhibit the growth of *C. albicans*, with an average IC_50_ value of 0.54 µg/ml, *T. interdigitale* (0.39 µg/ml) and *S. brevicaulis* (0.11 µg/ml). Ciclopirox olamine also had good antifungal activity, with the highest efficacy against dermatophytes (IC_50_ values between 1.02 and 2.62 µg/ml), followed by yeast (4.91 and 4.73 µg/ml) and mold (4.85 µg/ml; Table 2, also see Appendix A). Terbinafine was highly effective against the dermatophytes *T. rubrum* (0.0015 µg/ml), *T. soudanense* (0.0016 µg/ml), *T. interdigitale* (0.0038 µg/ml), and *E. floccosum* (0.0021 µg/ml), while significantly higher concentrations were required to kill the resistant strain *T. indotineae* (4.66 µg/ml; Table 2, also see Appendix A). In general, MIC values necessary for inhibition of dermatophytes, yeast, and mold were higher than IC_50_ values but followed the same trend. Two exceptions were noted, first, MIC values of sertaconazole nitrate in the case of *C. parapsilosis* were distinctly higher but were in line with the MIC values observed for *C. albicans*. Second, no inhibition of *C. albicans* was achieved with terbinafine in soluble amounts (Table 2). Compared to sertaconazole nitrate, higher concentrations of ciclopirox olamine (Table 2) were necessary to achieve an antifungal effect. The results for sertaconazole nitrate against *T. rubrum* and *T. soudanense* were comparable to those of terbinafine. However, terbinafine exhibited a higher efficacy against *T. interdigitale* and *E. floccosum*. Sertaconazole nitrate was significantly more effective against *T. indotineae*, *S. brevicaulis*, *C. albicans*, and *C. parapsilosis*. However, in this setting, it needed higher concentrations to be effective against *T. interdigitale* (Table 2, also see Appendix A).

**Table 2:** Antimicrobial activity of sertaconazole nitrate, ciclopirox olamine, and terbinafine according to MLN

	Sertaconazole nitrate		Ciclopirox olamine		Terbinafine	
	IC_50_ (µg/ml)	MIC (µg/ml)	IC_50_ (µg/ml)	MIC (µg/ml)	IC_50_ (µg/ml)	MIC (µg/ml)
*Trichophyton rubrum*	0.0028 ± 0.0003	1.3	1.02 ± 0.22	10.0	0.0015 ± 0.0002	0.006
*Trichophyton soudanense*	0.0014 ± 0.0001	3.1	1.43 ± 0.78	5.0	0.0016 ± 0.0005	0.015
*Trichophyton interdigitale*	0.39 ± 0.01	12.5	2.62 ± 0.62	10.0	0.0038 ± 0.0008	0.02
*Trichophyton indotineae*	0.018 ± 0.003	3.8	2.09 ± 0.78	10.0	4.66 ± 0.04	20.0
*Epidermophyton floccosum*	0.022 ± 0.005	1.6	1.46 ± 0.22	7.5	0.0021 ± 0.0001	0.02
*Scopulariopsis brevicaulis*	0.11 ± 0.03	7.5	4.85 ± 1.79	60.0	0.69 ± 0.03	40.0
*Candida albicans*	0.54 ± 0.12	40.0	4.01 ± 0.71	40.0	24.17 ± 2.99	n.d.
*Candida parapsilosis*	0.0025 ± 0.0001	20.0	4.73 ± 0.09	80.0	0.098 ± 0.002	3.8

*Note*: Dose–response curves yielded by MLN are presented in Appendix A.

Abbreviations: IC50, half‐maximal inhibitory concentration; MIC, minimum inhibitory concentration; MLN, microplate laser nephelometry; n.d., not determinable.

### 3.2 Antifungal treatment of artificially infected full skin models

**Figure 1:**
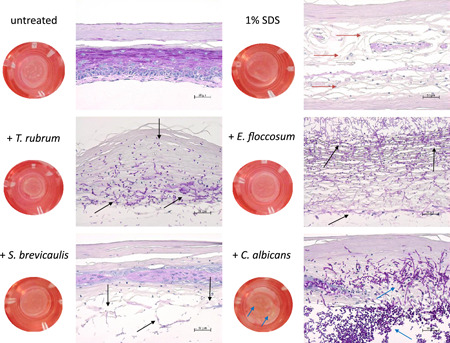
Macroscopic and microscopic images (PAS staining, 200‐fold magnification) after infection of the full skin models with *T. rubrum*, *E. floccosum*, *S. brevicaulis*, and *C. albicans* in comparison to the untreated and 1% SDS‐treated full skin model. The fungal outgrowth of dermatophytes and mold at 72 h is signified with black arrows. The yeast had spread widely in the skin model and pseudo mycelia growth was even macroscopically recognizable on the surface of the skin model (blue arrows). The control treatment with 1% SDS also resulted in significant damage to the full skin models (red arrows). PAS, periodic acid–Schiff; SDS, sodium dodecyl sulfate

**Figure 2:**
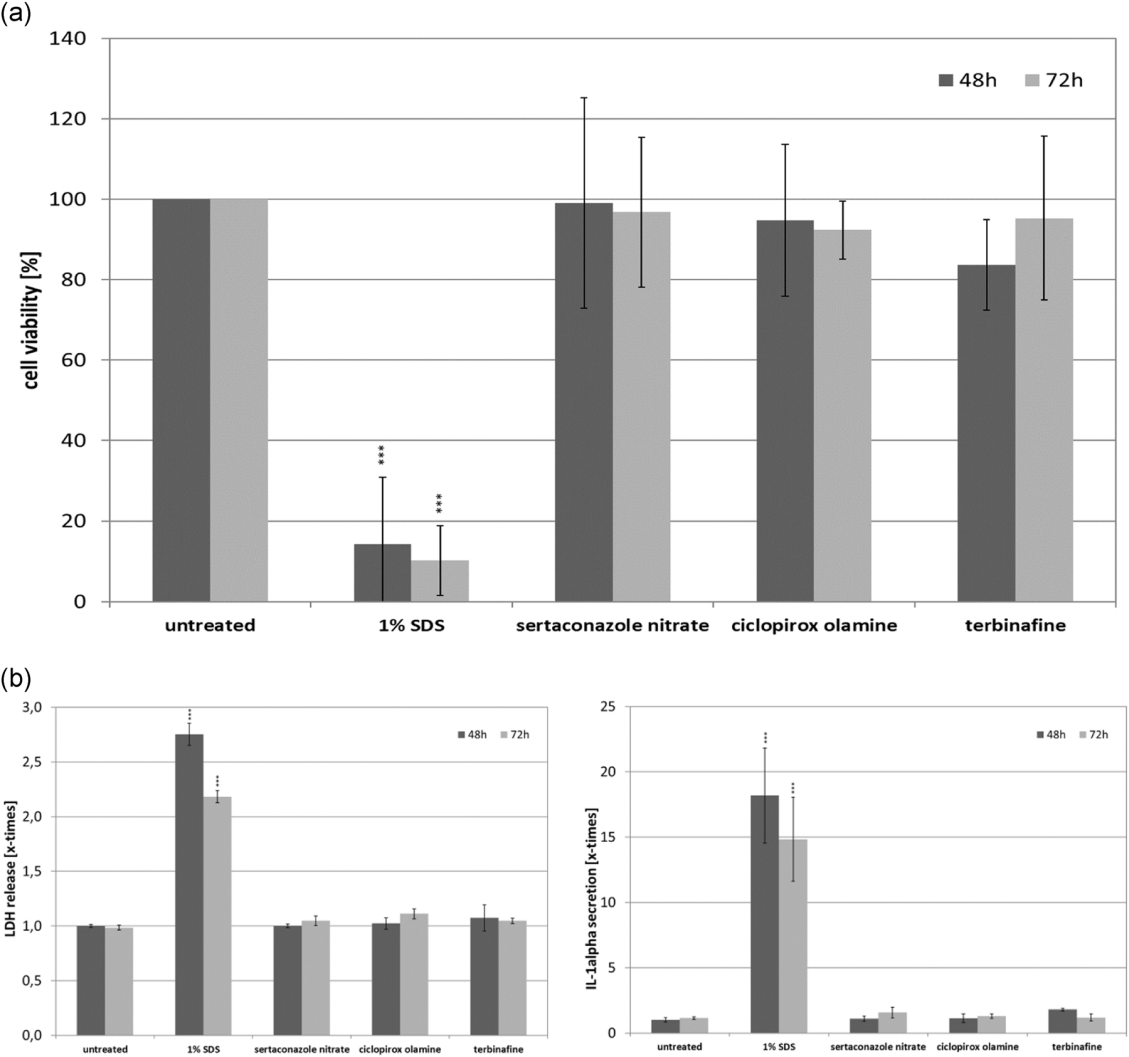
(a) Influence of sertaconazole nitrate, ciclopirox olamine, and terbinafine on the viability of the full skin models as well as the release of (b) LDH and (c) IL‐1α as a sign of damage to the full skin models. IL‐1α, interleukin 1α; LDH, lactate dehydrogenase

**Figure 3:**
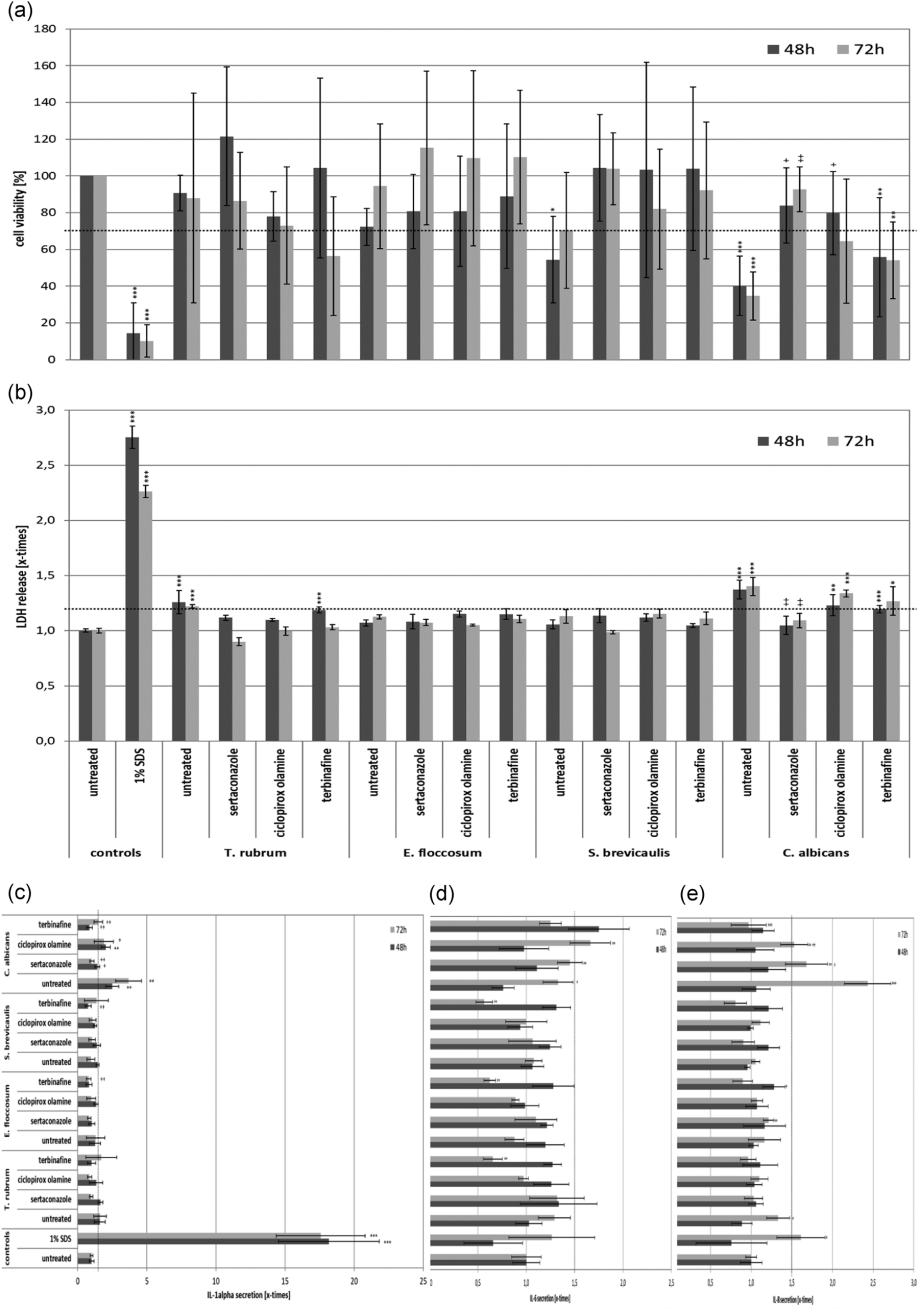
(a) Behavior of 3D skin models infected with *T. rubrum*, *E. floccosum*, *S. brevicaulis*, and *C. albicans* either untreated or after treatment with sertaconazole nitrate, ciclopirox olamine, or terbinafine, with regard to the viability of the cells in the full skin models. Release of (b) LDH and (c) IL‐1α as signs of damage to the full skin models, as well as monitoring of the secretion of the proinflammatory mediators (d) IL‐6 and (e) IL‐8. 3D, three‐dimensional; IL‐1α, interleukin 1α; LDH, lactate dehydrogenase

**Figure 4:**
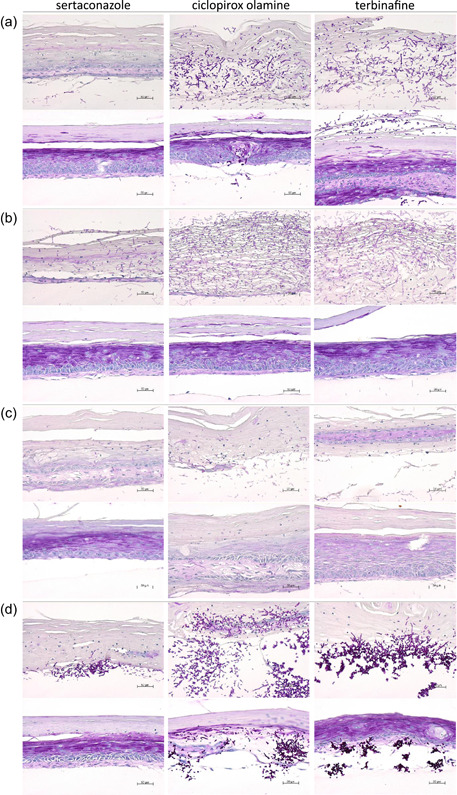
Histological results after infection of the full skin models with (a) *T. rubrum*, (b) *E. floccosum*, (c) *S. brevicaulis*, and (d) *C. albicans* and treatment with sertaconazole nitrate, ciclopirox olamine, or terbinafine after 72 h (PAS staining, 200‐fold magnification). PAS, periodic acid–Schiff

Growth of the resting spores of *T. rubrum*, *E. floccosum*, *S. brevicaulis*, and *C. albicans* in the full skin models was verified by PAS staining (Figure 1) and HE staining (Appendix B). For the two dermatophytes and the mold, fungal hyphae growth was visible after 48 h (Supporting Information Data) and a distinct increase in the fungal load was discernable after 72 h (Figure 1, black arrows). This resulted in indistinct changes of the dermal and epidermal skin layers caused by the keratolytic action of the fungi. It was found that *C. albicans* rapidly grew on the skin models. Already after 48 h, significant pseudohyphae outgrowth was discernable in most of the inspected sections (Appendix B) and after 72 h, the skin models were almost completely drawn through by pseudohyphae and aggregations of yeast cells (Figure 1, red arrows). The control treatment with 1% SDS resulted in significant damage to the full skin models (Figure 1, blue arrow). The concentrations of sertaconazole nitrate, ciclopirox olamine, and terbinafine selected (Table 1) had no negative effects on the viability of the full skin models (Figure 2a). Cell viability did not fall below the DIN EN ISO 10993‐5 threshold of 70%. Treatment with 1% SDS led to a significant drop in the viability of the skin models to approximately 20% (*p* < 0.001). No distinct release of LDH (Figure 2b) or IL‐1α (Figure 2c) was observed when using the sample concentrations compared to the untreated skin models and the SDS control. Infection of the skin models resulted in a biological relevant reduction of cell viability (<70% of the untreated control) in the case of *S. brevicaulis* and *C. albicans* (Figure 3a). This was accompanied by a significant increase in LDH release (Figure 3b; *p* < 0.001) and IL‐1α secretion (Figure 3c; *p* < 0.01) from *C. albicans*‐infected 3D skin models. Interestingly, no augmented generation of these markers was found after *S. brevicaulis* infection despite the decreased cell viability. Infection with the dermatophytes *T. rubrum* and *E. floccosum* did not affect 3D skin model viability but *T. rubrum* microconidia growth resulted in significantly increased LDH release (Figure 3b; *p* < 0.01). No other effects on these markers in the skin models were observed. Therefore, we also examined the secretion of IL‐6 (Figure 3d) and IL‐8 (Figure 3e) in the full skin models after infection. These inflammatory cytokines were also significantly induced by *C. albicans* after 72 h (*p* < 0.05/0.001). Treatment of *C. albicans* infected skin models with sertaconazole nitrate (Figure 3a) significantly increased cell viability at 48 (*p* < 0.05) and 72 h (*p* < 0.01) and decreased LDH release (Figure 3b) and IL‐1α secretion (Figure 4c) significantly compared to the infected skin models that were left untreated (*p* < 0.01). Terbinafine and ciclopirox olamine had only a marginal effect on skin model survival (Figure 3a) and were not able to reduce LDH release (Figure 3b). However, terbinafine seemed to exert an anti‐inflammatory effect in all cases, and decreased secretion of IL‐1α (Figure 3c), IL‐6 (Figure 3d), and partly IL‐8 (Figure 3e) was observed. Ciclopirox olamine was able to reduce IL‐1α release in infection with *C. albicans, E. floccosum*, and *T. rubrum* after 72 h (Figure 3c). Furthermore, under sertaconazole nitrate treatment the IL‐1α liberation by infected skin models declined to control levels, indicating an effective control of infection (Figure 3c). Release profiles for IL‐6 and IL‐8 showed no consistent changes with the treatments.

The assessment of the damage to the full skin models from infection and the effectiveness of the treatment were further carried out by evaluating the histological results (Figure 4, Appendix B). The evaluation was based on the identification of fungal hyphae or yeast cells and the general amount present in the examined sections as well as the observed keratolytic damage to the epidermal and dermal layer (see Supporting Information for further information). Sertaconazole nitrate mostly prevented the outgrowth of *T. rubrum* spores over 48 h and terbinafine yielded similar results (Appendix B). After 72 h, hyphae growth was noted in some instances but was distinctly less compared to the untreated control (Figure 4a). Moreover, ciclopirox olamine and terbinafine were able to decrease the growth of *T. rubrum* hyphae over 72 h, but to a much lesser extent than sertaconazole nitrate. In accordance, typical keratolytic damage to the dermal and epidermal layers was visible. The growth of *E. floccosum* in the skin models was also markedly decreased by sertaconazole nitrate over 48 h (Appendix B). The fungal load was still reduced at 72 h compared to the untreated control and skin models were less damaged (Figure 4b). Comparable results were achieved by ciclopirox olamine and terbinafine against *E. floccosum* infections in the 3D skin models. The test samples were further very effective against *S. brevicaulis* for up to 48 h (Appendix B). Evaluation after 72 h showed increased fungal hyphae loads under ciclopirox olamine treatment compared to sertaconazole nitrate and terbinafine, which demonstrated comparable efficacy against *S. brevicaulis* in the infected full skin models (Figure 4c). The growth of *C. albicans* in the infected full skin models was distinctly reduced by sertaconazole nitrate over 48 h (Appendix B) as well as over 72 h (Figure 4d). Here, treatment with ciclopirox olamine and terbinafine had distinctly lower effects.

## 4 DISCUSSION

Several antifungal drugs have been recommended for the treatment of dermatomycoses. However, it is still challenging to eliminate not only growing fungi but also fungal spores. Besides featuring a thick cell wall, fungal spores show minimal cell metabolism, which both reduces their susceptibility toward adverse environmental conditions, such as reduced oxygen content or antifungal therapy (Seidl et al., 2015). The main reasons for treatment failure and disease recurrence are a patient's failure to continue treatment (Behnam et al., 2020) and the formation of fungal spores (Seidl et al., 2015). Reports of drug‐resistant fungal strains are steadily increasing (Afshari et al., 2016; Ebert et al., 2020; Gupta et al., 2021; Mukherjee et al., 2003; Nenoff et al., 2020; Rudramurthy et al., 2018; Salehi et al., 2018; Yamada et al., 2017). Consequently, it is important to test the sporicidal effects, as well as the susceptibility of growing cells to old and new antifungal substances, using a variety of strains implicated in dermatomycoses.

The allylamine terbinafine is the best‐studied antifungal drug, with most studies using *T. rubrum* or *E. floccosum* for testing, but several studies have also investigated *T. indotineae*, *Candida* species, or *S. brevicaulis*. Terbinafine inhibits the enzyme squalene epoxidase, a crucial enzyme involved in ergosterol biosynthesis (Osborne et al., 2005). Inhibition of this enzyme leads to the accumulation of squalene and depletion of ergosterol inside the fungal cells, ultimately leading to cell death (Afshari et al., 2016). Terbinafine is highly effective against the dermatophytes *T. rubrum*, *T. soudanense*, *T. interdigitale*, and *E. floccosum*, findings that confirmed those of earlier studies with *T. rubrum* (Jessup et al., 2000; Mock et al., 1998; Singh et al., 2007) and *E. floccosum* (Esteban et al., 2005; Jessup et al., 2000; Salehi et al., 2018). Nevertheless, *T. indotineae* and *rubrum* strains with *Erg1* point mutations encoding for squalene epoxidase show an increase in terbinafine MIC_90_ values of several magnitudes (Ebert et al., 2020; Rudramurthy et al., 2018). *T. rubrum* is an anthropophilic species, and together with *T. interdigitale*, it is the most common cause of skin and nail infections worldwide (Nenoff et al., 2014; Seebacher et al., 2008). Before *T. rubrum* and *T. interdigitale*, *E. floccosum* was the most common pathogen associated with tinea pedis (Zhan & Liu, 2017). Currently, a high incidence of *Epidermophyton* spp. infections is only reported from countries such as Iran or Nigeria (Zhan & Liu, 2017). However, *E. floccosum* remains associated with chronic courses of dermatophytosis (Vineetha et al., 2018). Only one report of the MIC values of terbinafine against *T. soudanense* was found (Mock et al., 1998), and this study produced results similar to ours. *T. soudanense* is an anthropophilic dermatophyte that occurs mainly in the tropics, especially in sub‐Saharan Africa. Hence, infections with this dermatophyte are expected among travelers and immigrants from African countries and their contact persons, who require dermatological consultation (Nenoff et al., 2018). Silva et al. (2014) reported that slightly lower concentrations of terbinafine are needed to inhibit *T. interdigitale* growth than were identified in our study. However, higher concentrations were required in other studies (Behnam et al., 2020; Rudramurthy et al., 2018; Salehi et al., 2018). The differences between the MICs determined in different studies probably depend on the test methods, the growth conditions (e.g., incubation time) used, and the usage of fungal mycelia or spores. Moreover, the MIC depends strongly on the isolates selected for testing and their susceptibility. This was most distinctly demonstrated for *T. mentagrophytes*, for which the terbinafine‐resistant variant *T. indotineae* was tested here. Consistently higher concentrations were needed to kill the resistant isolate than previously reported (Badali et al., 2015; Behnam et al., 2020; Carrillo‐Muñoz et al., 2008; Esteban et al., 2005; Jessup et al., 2000; Mock et al., 1998; Singh et al., 2007). There is a veritable epidemic of chronic recalcitrant dermatomycoses due to *T. indotineae* in India (Ebert et al., 2020). However, these dermatophytes are now also increasingly identified in Germany and other European countries (Burmester et al., 2019; Nenoff et al., 2020; Süß et al., 2019). Some studies also reported higher MIC values for terbinafine against *T. rubrum*, which might be attributed to the heightened occurrence of resistant isolates (Badali et al., 2015; Carrillo‐Muñoz et al., 2008; Esteban et al., 2005; Rudramurthy et al., 2018; Salehi et al., 2018; Silva et al., 2014). Higher concentrations of terbinafine were also needed to kill the mold *S. brevicaulis* in this study than were found in previous reports (Carrillo‐Muñoz et al., 2008; Cuenca‐Estrella et al., 2003; Skóra et al., 2014). *Scopulariopsis* spp. are common soil saprophytes and have been isolated from a wide variety of substrates (Cuenca‐Estrella et al., 2003). They can cause superficial, subcutaneous, and invasive infections in humans that are very difficult to treat. *S. brevicaulis* is resistant to amphotericin B, flucytosine, and azole compounds in vitro without a prior history of antifungal treatment (Cuenca‐Estrella et al., 2003). No inhibition of *C. albicans* could be achieved with terbinafine in soluble amounts, a finding that is consistent with reports that terbinafine has little to no effect on *Candida* spp., although the filamentous form is thought to be susceptible. Hence, a wide effective concentration range is usually found with *Candida* spp. (Craik et al., 2017; Jessup et al., 2000; Ryder et al., 1998; Silva et al., 2014).

Ciclopirox olamine has been examined in a number of studies as a promising sporicidal agent. It acts through the chelation of polyvalent metal cations, leading to the inhibition of many cellular activities and modification of the fungal plasma membrane (Monti et al., 2019). It was believed to be the only antifungal drug with a sporicidal action as spores are resting cells and therefore do not synthesize ergosterol and, consequently, antifungal drugs that inhibit enzymes necessary for ergosterol production are ineffective (Seidl et al., 2015). However, only Schaller et al. reported a similar MIC as observed here against *T. rubrum* (Schaller et al., 2009), and mostly, lower concentrations were reported to kill *T. rubrum* (Rudramurthy et al., 2018; Singh et al., 2007), *T. interdigitale* (Rudramurthy et al., 2018), *T. mentagrophytes* (Singh et al., 2007), *E. floccosum* (Singh et al., 2007), *S. brevicaulis* (Skóra et al., 2014), and the yeasts *C. albicans* and *C. parapsilosis* (Craik et al., 2017; Figueiredo et al., 2007).

As data regarding sertaconazole are scarce, this study aimed to investigate its effects against some of the most common and increasingly prevalent causes of dermatomycoses. Like other azoles, sertaconazole compounds inhibit the synthesis of the cell membrane component ergosterol by inhibiting sterol 14α‐demethylase, which results in the accumulation of sterol precursors and the disruption of mycelial growth and replication (Croxtall & Plosker, 2009; Monod et al., 2019). However, at higher concentrations, sertaconazole has been found to bind directly to nonsterol lipids in the fungal cell membrane, leading to increased permeability of the wall and subsequent lysis of the mycelium (Croxtall & Plosker, 2009). In general, similar MICs against *T. rubrum* have been reported for sertaconazole (Carrillo‐Muñoz et al., 2011; Croxtall & Plosker, 2009; Rudramurthy et al., 2018). In the case of *T. interdigitale* (Croxtall & Plosker, 2009; Rudramurthy et al., 2018), *E. floccosum* (Carrillo‐Muñoz et al., 2011; Croxtall & Plosker, 2009), *C. albicans*, and *C. parapsilosis* (Croxtall & Plosker, 2009), lower MICs have been reported, whereas Carillo‐Munoz et al. (2011) recorded a higher MIC against *T. mentagrophytes* than was found in this study.

Drug resistance is an increasing problem that affects all antifungal agents, including azoles. However, there are still limited data currently available regarding fungal resistances to sertaconazole nitrate (Croxtall & Plosker, 2009). Our results provide evidence that sertaconazole nitrate has sporicidal effects. In general, spores show an increased resistance due to a thick proteinaceous coat, a relatively impermeable inner spore membrane, a low water content, and proteins that protect the DNA. Despite these properties, spores can be killed, when crucial spore proteins, the spore's inner membrane, and one or more components of the spore germination apparatus are damaged (Setlow, 2014). In addition to inhibiting the ergosterol synthesis, a major constituent of fungal cell wall membranes, sertaconazole nitrate can directly bind to non‐sterol lipids in the cell membrane (Agut, Palacin, Sacristan et al., 1992; Agut, Palacin, Salgado et al., 1992; Elewski, 1993), which leads to increased permeability of fungal cell membranes and thereby to a rapid leakage of key intracellular components including adenosine triphosphate. This direct membrane damaging effect was shown in vitro, where sertaconazole produces a dose‐dependent reduction of intracellular ATP levels in suspensions of *C. albicans* (Agut, Palacin, Salgado et al., 1992). The possibility to assert this damage also on spore membranes, differentiating sertaconazole nitrate from other membrane‐active antifungals, could be conferred by the lipophilic benzothiophene ether of sertaconazole nitrate, which increases permeation capability through protein‐rich layers (Pfaller & Sutton, 2006). In addition, it is also conceivable that sertaconazole nitrate is adsorbed at or into the cell wall and further absorbed into the cell and inhibiting ergosterol biosynthesis from within. Seidl et al. (2015) postulated this additional mode of action, leading to the maintenance of the resting state due to insufficient ergosterol synthesis. The sporicidal activities of terbinafine, ciclopirox olamine, and sertaconazole nitrate were further examined using a 3D skin model artificially infected with *T. rubrum*, *E. floccosum*, *S. brevicaulis*, or *C. albicans*. Several studies on mostly bacterial, but also fungal, colonization using 3D epidermal models consisting of differentiated keratinocytes on an artificial membrane have been published so far (de Breij et al., 2012; Lerebour et al., 2004; Müller et al., 2014; Shepherd et al., 2009; Son et al., 2014). Some also used 3D full skin models, featuring a dermal layer with fibroblasts and an epidermal layer with differentiated keratinocytes, as they are present in human skin, to reflect a more in vivo‐like situation for infection studies (Haisma et al., 2014; Holland et al., 2009, 2008; Kitisin et al., 2020; Kühbacher et al., 2017; Popov et al., 2014). Recently, such a 3D skin model was successfully employed to investigate the antiseptic treatment of *S. aureus* infections in a skin‐like environment (Reddersen et al., 2019) and to explore the effects of *T. benhamiae* contagion on human skin cells in vitro (Hesse‐Macabata et al., 2019). To the best of our knowledge, this is the first report on artificial infection of a 3D skin model with different types of fungal spores and subsequent treatment with antifungal agents.

Forty‐eight hours after inoculation of the spores into the lower dermal layers, the yeast had spread widely and hyphae growth was even macroscopically recognizable on the surface of the skin model. This infection was accompanied by a reduction in cell viability and a significant increase in LDH release as well as an upsurge in the secretion of proinflammatory cytokines. The destructive effects of *T. rubrum*, *E. floccosum*, and *S. brevicaulis* on skin model viability and damage marker liberation were much less pronounced, and fungal spread was mainly evaluated using histological approaches. Consistent with the MLN results, it was found that sertaconazole nitrate reduced the outgrowth of *T. rubrum* spores, prevented the progression of *E. floccosum* and *S. brevicaulis* hyphae growth, and reduced the expansion of *C. albicans* pseudohyphae in the infected full skin models. Ciclopirox olamine only demonstrated comparable effectiveness against *E. floccosum* and reduced the spread of *T. rubrum* and *S. brevicaulis*, but could not prevent hyphae growth and damage to the skin models. It further exerted a distinctly lower effect on *C. albicans* compared to sertaconazole nitrate. Terbinafine exhibited comparable effectiveness to sertaconazole nitrate against *E. floccosum* and inhibited to a lesser extent *S. brevicaulis* growth. It further had a lower effect on *C. albicans* growth compared to sertaconazole nitrate but failed to achieve a similar reduction of *T. rubrum*. As only a small number of fungal strains were investigated and the number of tests was limited, the results must be interpreted with caution.

In summary, these findings demonstrate the necessity of testing antifungal agents under in vivo‐like conditions to evaluate their efficacy. Several studies stressed that fungal spores are the primary cause of infection with dermatophytes (Aljabre et al., 1992; Rashid et al., 1995; Seidl et al., 2015). Consequently, only an antifungal with sufficient activity against the resting bodies of fungi and yeasts can deliver sufficient results to achieve acceptable long‐term cure rates. Translated into daily practice, this would mean that treatment of recurring dermatomycoses with sporicidal substances might efficiently prevent treatment failures caused by the persistence of fungal spores. This study showed that sertaconazole nitrate can inhibit the growth of dermatophytes, molds, and yeasts. It further reduced the growth of fungal and yeast spores over 72 h in infected 3D skin models. Ciclopirox olamine exhibited good antifungal activity, although higher concentrations were needed compared to sertaconazole nitrate and the efficacy in the infected 3D skin models was lower, especially for the yeast. Terbinafine was highly effective against most dermatophytes and inhibited the growth of *E. floccosum* as well as *S. brevicaulis* in the 3D skin model, but higher concentrations were required to kill the resistant strain *T. indotineae*. In addition, terbinafine displayed significantly lower treatment efficacy in the *Candida* spp. infections. In conclusion, sertaconazole nitrate might have advantages over the commonly used antifungals ciclopirox olamine and terbinafine in combating persistent spores and thus for treating recurring dermatomycoses.

## CONFLICT OF INTERESTS

Claudia Neumeister and Marcus Rudolf Götz are employees of Dr. Pfleger Arzneimittel GmbH, Bamberg, Germany. The sertaconazole nitrate studied is commercially distributed by Dr. Pfleger Arzneimittel GmbH.

## ETHICS STATEMENT

None required.

## AUTHOR CONTRIBUTIONS

**Sarah Fink**: Conceptualization (supporting), formal analysis (equal), Writing – original draft (supporting), writing – review and editing (supporting). **Anke Burmester**: Conceptualization (supporting), formal analysis (equal), writing – original draft (supporting), writing – review and editing (supporting). **Uta‐Christina Hipler**: Conceptualization (lead), writing – review and editing (supporting). **Claudia Neumeister**: Conceptualization (supporting), writing – original draft (supporting), writing – review and editing (supporting). **Marcus Rudolf Götz**: Conceptualization (supporting), writing – original draft (supporting), writing – review and editing (supporting). **Cornelia Wiegand**: Conceptualization (lead), formal analysis (supporting), writing – original draft (lead), writing – review and editing (lead).

## Figures and Tables

**Figure A1:**
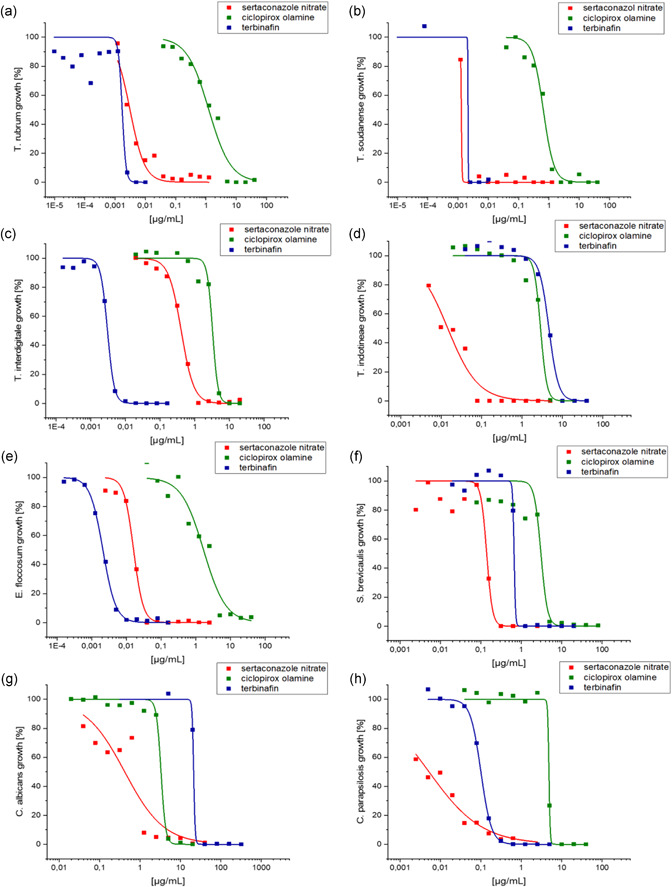
Comparison of the dose–response curves of sertaconazole nitrate (red squares and line), ciclopirex olamine (green squares and line), and terbinafine (blue squares and line) against (a) *T. rubrum*, (b) *T. soudanense*, (c) *T. interdigitale*, (d) *T. indotineae*, (e) *E. floccosum*, (f) *S. brevicaulis*, (g) *C. albicans*, and (h) *C. parapsilosis*

**Figure A2:**
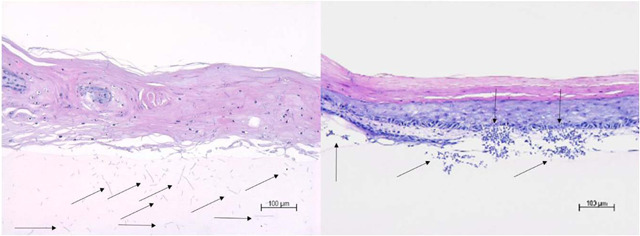
Fungi and yeast can be distinguished as dark blue hyphae and blastospores in the HE‐stained sections (see the following figure). Two complete sections per three‐dimensional skin model were evaluated according to the following parameters (refer to the first table below) and results can be gathered from the second table below. On the following pages, representative pictures for each section of the skin models and independent experiments are given. Dark blue stained hyphae and blastospores in the HE‐stained section are indicated by black arrows. HE, hematoxylin and eosin

## Data Availability

All data are presented in the article and its appendices.

## APPENDIX A ASSESSMENT OF ANTIMYCOTIC ACTIVITY USING MICROPLATE LASER NEPHELOMETRY

See Figures  A1 and A2, Tables A1 and A2.

**Table A1:** Description of the evaluation parameters and the assigned value

Sign	Description
0	No fungal presence detected, skin model structure appears undisturbed
+	Slight hyphae/blastospore growths, separation at the dermal layer due to effects of the fungi/yeast
++	Hyphae/blastospore growth visible, damage to the dermal or epidermal layer of the skin model visible due to fungi/yeast growth
+++	Distinct hyphae/blastospore growth discernable (often complete overgrowth of the skin model), significant damage to the dermal or epidermal layer of the skin model visible due to keratolytic action of fungi/yeast

## APPENDIX B EVALUATION OF FUNGAL AND YEAST GROWTH IN THE THREE‐DIMENSIONAL SKIN

**Table A2:** Evaluation of fungal and yeast growth in the untreated and treated three‐dimensional skin models

		Untreated control				Sertaconazol nitrate				Ciclopirox olamine				Terbinafine			
	48 h	+	+	+	+	+	+	0	0	++	+	+	+	+	0	+	0
TR		++	+	+	+	0	+	0	0	+	+	+	+	+	0	+	0
	72 h	++	++	++	+++	0	++	0	+	++	+++	+	+	+++	++	+++	+
		++	++	++	++	0	+	0	++	++	+++	+	+	+++	+++	+++	+
	48 h	++	++	+	+	0	+	+	++	++	++	++	+	++	++	++	+
EF		++	++	+	+	+	0	+	+	++	++	++	0	++	++	++	0
	72 h	+	+++	+++	+++	+	+++	++	0	+	+++	+++	0	+	+++	+++	0
		+	+++	+++	+++	+	++	++	0	+	+++	+++	0	0	+++	+++	0
	48 h	++	+	+	+	++	0	0	0	++	0	+	0	++	+	0	0
SB		++	+	+	++	0	0	0	0	0	0	0	+	++	+	+	0
	72 h	++	++	+	+++	++	+	0	0	+++	++	++	+	+	+	+++	0
		++	++	++	+++	+	++	+	0	++	+	++	++	++	+	++	0
	48 h	+++	++	++	+	+	0	++	++	++	+++	++	++	+++	++	++	++
CA		++	+	++	++	+	0	+	+	++	+++	++	0	+++	++	++	+
	72 h	+++	+++	+++	+++	++	++	0	++	+++	+++	++	++	+++	++	++	+
		+++	+++	+++	+++	++	++	0	++	+++	+++	++	++	+++	++	++	++

Abbreviations: CA, *C. albicans*; EF, *E. floccosum*, SB, *S. brevicaulis*; TR, *T. rubrum*.
